# Changes in Epidemiological Characteristics in Children with *Mycoplasma pneumoniae* Seropositivity in Southwest China from 2022 to 2023

**DOI:** 10.3390/jcm15103655

**Published:** 2026-05-09

**Authors:** Zhengxiang Gao, Yifei Duan, Yu Wu, Yu Gou, Fan Yu

**Affiliations:** 1 Department of Laboratory Medicine, West China Second University Hospital, Sichuan University, Chengdu 610041, China; gaozx84@163.com (Z.G.); dyf2021@163.com (Y.D.); wy18008278520@163.com (Y.W.); 396590035@163.com (Y.G.); 2 Key Laboratory of Birth Defects and Related Diseases of Women and Children, Sichuan University, Ministry of Education, Chengdu 610041, China

**Keywords:** *Mycoplasma pneumoniae*, epidemiology, acute respiratory tract infections, children, China

## Abstract

**Background**: *Mycoplasma pneumoniae* (MP) is an important pathogen responsible for community-acquired respiratory infections in children. Global surveillance during the COVID-19 pandemic revealed a marked decline in MP activity. However, beginning in early summer 2023, multiple regions across China reported an unexpected resurgence of MP infections, highlighting the need for detailed epidemiological analysis. **Objective**: This study aimed to characterize the epidemiological features of MP seropositivity among children in Chengdu, southwest China, and to compare its patterns between the COVID-19 pandemic and post pandemic periods. **Methods**: A retrospective analysis was conducted on MP testing data from 39,552 children with acute respiratory infections who were treated at West China Second University Hospital, Sichuan University, between January 2022 and December 2023. **Results**: Both the number of MP tests conducted and the seropositivity rate were significantly lower during the pandemic period than during the post pandemic phase. Compared with male children, female children were more susceptible to MP seropositivity. In terms of age distribution, seropositivity rates were highest among toddlers (1–3 years) and school-aged children (6–14 years). During the pandemic period (2022), MP antibody-positive cases were observed mainly between January and July, whereas in the post pandemic phase (2023), the epidemic peak shifted from June to December. **Conclusions**: In this single-centre study in Chengdu, nonpharmaceutical interventions (NPIs) implemented during the COVID-19 pandemic was associated with a marked reduction in MP transmission. After these restrictions were lifted, a rebound in MP antibody positivity was observed among children in Chengdu, compared to the NPI period (2022), the post-NPI period (2023) showed a later seasonal peak, which may represent a delayed return to pre-pandemic patterns. Continuous strengthening of MP surveillance is necessary to provide early warning of potential resurgences and outbreak risks.

## 1. Introduction

Acute respiratory infections (ARIs) are major causes of childhood morbidity and mortality worldwide. Their high prevalence and incidence make ARIs a significant global child health concern [1]. According to statistics from the World Health Organization (WHO), ARI is the fourth leading cause of death worldwide. This common disease poses a serious threat to child health and imposes a substantial burden on healthcare systems in developing countries, including China [2]. Globally, hospitalizations of children due to ARIs remain persistently high, exceeding 12 million annually in some years. ARIs account for approximately 6% of the global disease burden—which is greater than that of some cancers and cardiovascular diseases—and pose a major challenge to healthcare systems worldwide [3]. The aetiology of paediatric ARI is complex and involves a wide range of pathogens, including bacteria, viruses, and atypical organisms. The substantial overlap in clinical manifestations caused by different pathogens presents major challenges for rapid aetiological diagnosis in clinical practice. In China, regional differences in environmental conditions, climate, and living habits lead to marked geographic variation in respiratory pathogen profiles, further complicating the prevention and control of paediatric ARIs [2]. *Mycoplasma pneumoniae* (MP) is a major pathogen responsible for ARI in children and adolescents worldwide. MP infection presents a broad clinical spectrum, ranging from mild upper respiratory tract infections to severe *MP* pneumonia (MPP). Furthermore, MP infection may be complicated by bronchitis and diverse extrapulmonary manifestations, posing a serious threat to child health and becoming life-threatening in severe cases [4].

The emergence and global spread of coronavirus disease 2019 (COVID-19) profoundly altered the pre-existing epidemiological landscape. During the early stages of the pandemic, strict implementation of nonpharmaceutical interventions (NPIs) was associated with a marked reduction in the circulation of common respiratory pathogens. Detection rates of MP, a common cause of acute respiratory infections in school-aged children and adolescents, experienced a dramatic decline during this period. Following the relaxation of containment measures and NPIs in early 2023, an unusual resurgence and off-season circulation of multiple respiratory pathogens was observed worldwide. This shift was accompanied by changes in epidemiological characteristics, with significant geographic and interinstitutional variation [5]. In this context, a marked resurgence of MP seropositivity posed a substantial threat to the paediatric population [4,6]. Subsequent data confirmed renewed MP outbreaks across Europe and Asia in 2023 [7]. The average detection rate sharply increased to 4.12% between April and September 2023, indicating the onset of a new epidemic cycle [7,8]. Notably, a unique “delayed resurgence” pattern emerged: between April 2022 and March 2023, the global MP positivity rate remained as low as 0.82% [9]. However, starting from April 2023, an unexpected rebound occurred across multiple continents. In Denmark, a nationwide MP epidemic broke out between October and December 2023, with positivity rates rising sharply [10]. In France, the ORIGAMI multicentre study reported 969 hospitalized MP cases, of which 6% required intensive care [11]. Similar resurgences were also documented in the Netherlands [12] and the United States [13].The epidemiological situation in China mirrored global trends. A sharp increase in paediatric respiratory disease cases was observed from October to December 2023, with MP identified as one of the predominant pathogens during this period [14]. Surveillance data highlighted a heavy MP burden: detection rates in Ningbo reached 23.12% among patients, and the MP positivity rate during the non-NPI phase (34.28%) was significantly higher than that during the NPI phase (6.35%) [15]. A study revealed that the positivity rate of MP began to increase significantly in May 2023, representing more than twice the peak number of cases during its outbreak in 2019 in southern China [16]. Against this backdrop of widespread MP activity, key information on the local incidence and evolving epidemiological patterns of MP-associated paediatric ARI remains limited. This knowledge gap highlights significant health risks for children, who remain vulnerable to unpredictable pathogen outbreaks.

Our research group previously analysed and published findings on the epidemiological characteristics of respiratory viruses in paediatric ARIs before and during the COVID-19 pandemic [5,17]. However, few studies have investigated the epidemiological features of MP infection in children during the post-COVID-19 restriction period. In particular, changes in the incidence, age distribution, and seasonal patterns of paediatric MP infection after the lifting of containment measures remain poorly characterized. To address this knowledge gap, we conducted a retrospective analysis of paediatric patients tested for *MP* at our hospital from January 2022 to December 2023. The aim of this study was to systematically elucidate the local epidemiological trends and clinical characteristics of MP infection in children with ARI. The findings are expected to inform age- and season-specific prevention strategies and provide a scientific basis for improved diagnosis, treatment, and public health interventions.

## 2. Materials and Methods

### 2.1. Study Population

We retrospectively analysed the data of children with suspected ARTIs who presented to West China Second University Hospital, Sichuan University, from 1 January 2022 to 31 December 2023. The inclusion criteria were as follows: (1) presented with clinical symptoms and signs consistent with an ARTI (e.g., cough, fever, rhinorrhoea, sore throat) as per standard paediatric criteria; and (2) had a clinician-documented diagnosis of ARTI (upper or lower) in their medical record. The exclusion criterion was a lack of information on ARTIs in the top three outpatient or discharge diagnoses. All patients were examined and diagnosed by clinicians. All patients were examined and diagnosed by clinicians. This study used 8 January 2023 (the date when major non-pharmaceutical interventions were officially lifted in Chengdu) as the cutoff, defining 2022 as the pandemic period and 2023 as the post-pandemic period. This classification was made for analytical convenience and does not imply an instantaneous binary change in control policies or population behaviors.

### 2.2. Sample Evaluation

Serum was collected from patients and sent to the hospital laboratory for testing. Meanwhile, the patients who had multiple visits for MP testing during 2022–2023, only the result from their first test was included. After receiving the serum samples, laboratory technicians immediately processed and tested the samples. MP IgM antibodies were detected by a MP-IgM CLIA microparticle assay (Autobio Diagnostics Co., Ltd., Zhengzhou, China). The MP antibody titre was measured using a commercial passive agglutination assay kit (Zhuhai Livzon Reagent Co., Ltd., Zhuhai City, China). The MP IgM > 1.0 S/CO and antibody titre ≥ 1:160, indicating a positive result. Both CLIA and passive agglutination performed on all samples.

### 2.3. Data Collection

The laboratory test results and patient demographic data (name, sex, age, clinical diagnosis, and sampling time) were extracted from the laboratory information system (LIS) of West China Second Hospital of Sichuan University. The study protocol was approved by the ethics committee of this hospital. The approval number: Medical Research Ethics Approval (290) of 2023.

## 3. Statistical Analysis

Statistical analysis was performed using SPSS 26.0 software. Categorical variables are expressed as numbers and percentages (*n*, %). Continuous variables (e.g., Age) were first tested for normality; categorical variables are expressed as numbers and percentages. Monthly MP positivity rates were calculated as (positive cases/total tests in that month) × 100 for each year. To compare rates between 2023 and 2022 for the same calendar month, we used two-proportion z-tests. For months with expected cell counts < 5, Fisher’s exact test was applied. Results are presented as rate ratios (RR) with 95% confidence intervals (CI) and *p* values. Owing to the unequal population size and data differences, the chi-square test or Fisher’s exact test was used to compare differences between groups (sex, and age groups), and *p* < 0.05 was considered to indicate statistical significance. To control for potential confounding factors and evaluate the independent association between year (2023 vs. 2022) and MP seropositivity, a multivariable binary logistic regression analysis was performed. The dependent variable was MP IgM positivity (positive = 2, negative = 1). The independent variables included: year (coded as 1 for 2022 and 2 for 2023, treated as a continuous variable), age group (categorical: 1 = 3–6 years, 2 = 29 days–1 year, 3 = 1–3 years, 4 = 0–28 days, 5 = 6–14 years, 6 = ≥14 years; with 3–6 years as the reference category), sex (1 = female, 2 = male; female as reference), patient type (1 = outpatient, 2 = inpatient; outpatient as reference), and month (1 = January, 2 = February, …, 12 = December; January as reference). All variables were entered simultaneously using the Enter method. Results are presented as adjusted odds ratios (OR) with 95% confidence intervals (CI) and *p* values.

## 4. Results

### 4.1. Population Characteristics

**Figure 1:**
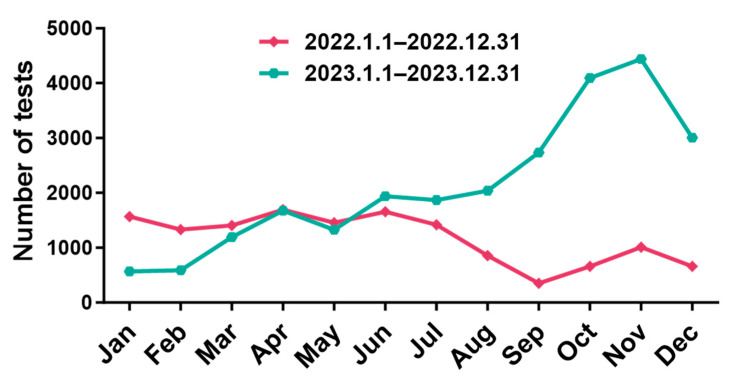
Monthly activity patterns of paediatric patients with acute respiratory infections underwent *Mycoplasma pneumoniae* testing between.

Table 1 and Figure 1 summarize the sociodemographic characteristics associated with all the collected samples. A total of 39,552 paediatric patients with ARIs underwent MP testing between 2022 and 2023, including 14,074 cases in 2022 and 25,478 in 2023. Children aged 3–6 years accounted for the largest proportion of tests in both years, followed by those aged 6–14 years, while neonates aged 0–30 days accounted for the smallest proportion. In 2022, 5926 patients (42.11%) were aged 3–6 years, and 8 were neonates (0.06%). In 2023, 10,380 (40.74%) were aged 3–6 years, and 11 were neonates (0.04%). In 2022, there were 12,054 male and 6603 female patients (ratio 1.82:1), while in 2023, there were 13,059 males and 12,419 females (ratio 1.05:1). The number of children older than 28 days tested was significantly greater in 2023. Outpatient cases predominated: in 2022, 12,054 (85.65%) were outpatients and 2020 (14.35%) were inpatients (ratio 5.97:1); in 2023, 23,302 (91.46%) were outpatients and 2176 (8.54%) were inpatients (ratio 10.7:1). With respect to month distribution, April 2022 had the highest testing volume (1692), and September had the lowest volume (354); in 2023, November had the highest (4438) and January had the lowest volume (567). The number of tests was higher in January–May 2022 than in 2023, but the number was higher in June to December 2023 than in 2022 (Figure 1).

### 4.2. Age Distribution of MP-Seropositive Paediatric Patients

**Figure 2:**
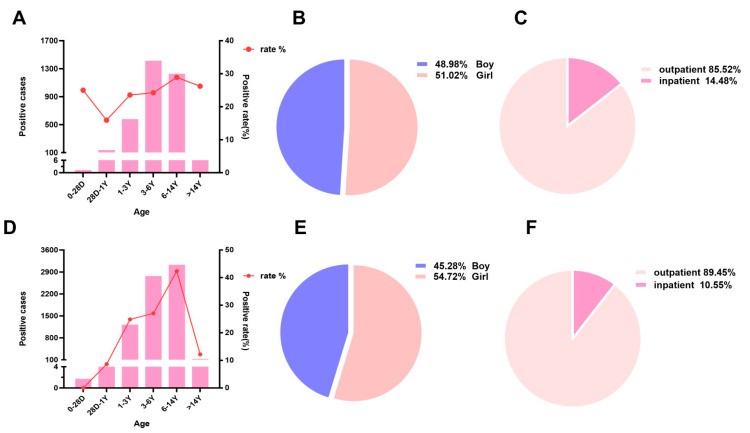
The demographic characteristics of the mycoplasma pneumoniae. (**A**–**C**) The proportions of different ages, sex and category of positive children in 2022. (**D**–**F**) The proportions of different ages, sex and category of positive children in 2023.

As shown in Table 2 and Figure 2A,D, the age-specific MP positivity rates differed between 2022 and 2023. In 2022, the highest positivity rate was observed in children aged 6–14 years (28.90%), followed by those >14 years (26.21%) and children aged 3–6 years (24.23%). The lowest rate was found in neonates (0–28 days, 25.0%, based on very small numbers). In 2023, the 6–14 years group still had the highest positivity rate (42.25%), followed by the 3–6 years group (27.10%). Notably, the positivity rate in the >14 years group dropped to 12.43%, and the 28 days–1 year group decreased from 15.92% in 2022 to 8.90% in 2023 (*p* < 0.001). The 1–3 years group showed a modest increase (23.55% in 2022 vs. 24.95% in 2023), but the difference was not statistically significant (*p* = 0.18). The neonatal group (0–28 days) also showed no significant difference between the two years (*p* = 0.72). In 2022, there were 3516 MP-positive cases and 10,560 negative cases, yielding a positivity rate of 24.98%. In 2023, there were 7520 positive cases and 17,958 negative cases, yielding a positivity rate of 29.54%. Among these, there were 1722 positive cases in males and 1794 in females in 2022 (Figure 2B); in 2023, there were 3405 positive cases in males and 4115 in females (Figure 2E). In 2022, outpatients accounted for 3006 positive cases (21.36% of the total tested in 2022), and inpatients accounted for 509 positive cases (3.62%) (Figure 2C). In 2023, outpatients accounted for 6727 positive cases (26.40% of the total tested in 2023), and inpatients accounted for 793 positive cases (3.11%) (Figure 2F).

### 4.3. Monthly Comparison of MP Positivity Rates

**Figure 3:**
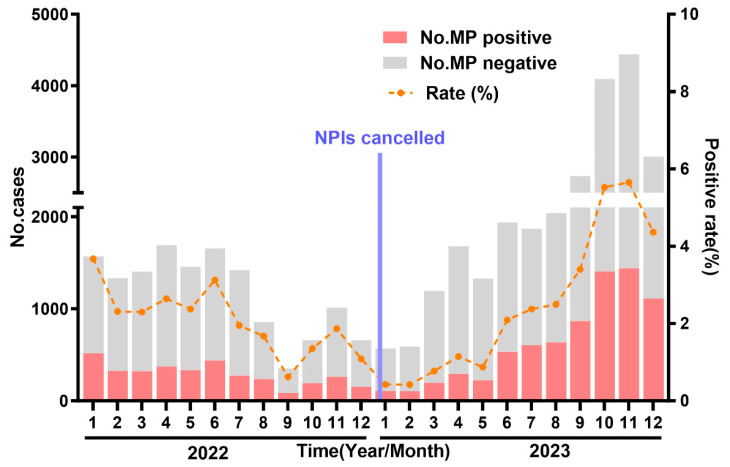
Monthly distribution of MP in paediatric ARTIs in Chengdu form January 2022 to December 2023. Red bars: MP-positive specimens; Gray bars: MP-negative specimens; Line: MP positivity rate. MP: Mycoplasma pneumoniae; ARIs: Acute respiratory infections.

To better assess seasonal changes, we compared monthly MP positivity rates between 2022 and 2023 (Figure 3 and Table 3). In 2022, the positivity rate peaked in January (32.97%), gradually declined, showed a modest increase in June (26.54%) and August (27.54%), then decreased to the lowest level in September (24.58%), and remained between 23% and 29% from October to December. In 2023, the positivity rate was low in January (19.05%), remained between 16% and 18% from February to May, then increased steadily from June onward, reaching 32.35% in July and peaking at 36.94% in December. Compared with 2022, the positivity rates from January to May 2023 were significantly lower (RR 0.578–0.791, all *p* < 0.01). The rate in June 2023 was similar to June 2022 (RR = 1.036, *p* = 0.527). From July onward, all monthly positivity rates in 2023 were higher than those in 2022: July (RR = 1.677, *p* < 0.001), August (RR = 1.131, *p* = 0.055, borderline significant), September (RR = 1.289, *p* = 0.010), October (RR = 1.186, *p* = 0.010), November (RR = 1.249, *p* < 0.001), and December (RR = 1.594, *p* < 0.001).

### 4.4. Multivariable Logistic Regression Analysis

To assess whether the year 2023 was independently associated with higher MP seropositivity after adjusting for potential confounders, a multivariable binary logistic regression analysis was performed. As shown in Table 4, after adjusting for age group, sex, patient type, and month, the year 2023 remained significantly associated with higher MP seropositivity (adjusted OR = 1.120, 95% CI: 1.063–1.180, *p* < 0.001). Inpatients had a higher positivity rate than outpatients (OR = 1.331, 95% CI: 1.236–1.434, *p <* 0.001). Males had a lower positivity rate than females (OR = 0.714, 95% CI: 0.682–0.747, *p* < 0.001), indicating that female children were at higher risk.

Regarding age groups (with 3–6 years as reference), the 6–14 years group showed the highest positivity rate (OR = 1.635, 95% CI: 1.552–1.723, *p* < 0.001). The other age groups all had lower positivity rates than the 3–6 years group: 29 days–1 year (OR = 0.379, 95% CI: 0.331–0.434, *p* < 0.001), 1–3 years (OR = 0.915, 95% CI: 0.859–0.976, *p* = 0.007), and ≥14 years (OR = 0.356, 95% CI: 0.307–0.413, *p* < 0.001). The 0–28 days group was not significantly different from the reference group (OR = 0.344, 95% CI: 0.078–1.523, *p* = 0.160).

For month (with January as reference), positivity rates from February to July were significantly lower (OR ranging from 0.555 to 0.831, all *p* < 0.01), while October and December showed significantly higher rates (October: OR = 1.157, *p* = 0.015; December: OR = 1.204, *p* = 0.003). No significant differences were observed for August, September, or November compared to January (all *p* > 0.05). These findings indicate an autumn–winter peak (October–December) of MP circulation.

## 5. Discussion

According to the latest Global Burden of Disease (GBD) report, ARI remains among the leading causes of years of healthy life lost among children worldwide [18,19]. To elucidate the characteristics of the paediatric ARI pathogen spectrum in this region, paediatric clinical samples were prospectively collected from January 2022 to December 2023 at West China Second University Hospital, Sichuan University, a major medical centre for women and children in western China. Children are particularly susceptible to respiratory pathogens because of their developing respiratory and immune systems, as well as generally poor adherence to personal protective measures such as mask wearing. Paediatric respiratory infections involve various pathogens, among which viruses and atypical organisms predominate. In patients with ARI, MP has been identified as an important pathogen, most commonly causing contagious respiratory illnesses [20,21].

Following the first report of the COVID-19 outbreak caused by SARS-CoV-2 in Wuhan, China, at the end of 2019, a series of NPIs were rapidly implemented nationwide, effectively curbing viral transmission. Notably, while these NPIs effectively controlled COVID-19, they also altered the epidemiological patterns of other atypical respiratory pathogens. Comparative analysis revealed that compared with those during the pandemic period, both the number of MP tests and the number of seropositive detections increased significantly during the post pandemic period. With the exception of neonates (0–28 days), the MP seropositivity rate was markedly higher across all the other paediatric age groups than during the pandemic. This phenomenon may be partly attributed to the stringent infection control and case management policies implemented during the pandemic, which substantially suppressed the community transmission of pathogens. Conversely, limitations in surveillance systems—specifically, the failure to test a large number of symptomatic individuals—may have led to an underestimation of positivity rates [22]. This consideration also applies to the interpretation of MP antibody positivity rates. Previous studies have confirmed that the detection rate of MP during the NPI phase was significantly lower than that during the non-NPI phase, which is consistent with the findings of the present study [15].

On the basis of our analysis, during the COVID-19 pandemic and the post-pandemic period, children aged 3–6 years and 6–14 years were the most susceptible to MP seropositivity among the six age groups, which is consistent with previous reports [23,24]. Compared with infants and toddlers, preschool-aged and school-aged children spend more time in group settings such as schools, increasing their opportunities for pathogen exposure. Enclosed and crowded environments further increase the risk of MP transmission and infection [25]. Although some studies have suggested a possible role of declining maternal antibody levels with age [26]. It should be noted that we did not directly measure exposure patterns; therefore, this interpretation remains speculative.

This study revealed that the MP seropositivity rate was higher among female patients than among male patients, both during and after the pandemic, which is consistent with the findings of previous studies [23,27]. Although the association between female sex and MP seropositivity remained significant after adjustment, given the retrospective observational design and the possibility of unmeasured confounding (e.g., healthcare-seeking behaviour, testing thresholds), this finding should not be interpreted as evidence of intrinsic biological susceptibility in females. Future prospective studies incorporating behavioural, immunological data and PCR confirmation are needed to explore potential mechanisms. This phenomenon suggests that females may be more prone to MP antibody positivity, potentially related to behavioural and social differences between the sexes. Conversely, the MP seropositivity rate was relatively low among infants under 1 year of age and individuals older than 14 years. Almost no positive cases were detected among neonates aged 0–28 days, which may be attributed to the very limited social contact and low exposure opportunities at this stage of life. The MP positivity rate in individuals over 14 years of age was less than 1%, differing from the 11.05% detection rate reported in some adult studies [28]. This discrepancy may be due to the study being conducted in a women’s and children’s hospital, resulting in selection bias and deviation from the general epidemiological characteristics of adults.

This study observed significant differences in the seasonal pattern of MP positivity rates between 2022 and 2023. In 2022 (during strict implementation of NPIs), the positivity rate peaked in January (32.97%) and June (26.54%), showing a predominantly winter–spring pattern. In contrast, in 2023 (after NPIs were lifted), the positivity rate began to rise steadily from June onwards, was significantly higher than the 2022 levels from July to December, and reached its highest peak in December (36.94%). Previous studies have shown that in southern China, MP infections in non-pandemic years typically peak in summer and autumn [29]. The sustained peak from July to December 2023 likely reflects the combined effect of accumulation of susceptible children and pathogen transmission rebound following the lifting of NPIs. Notably, the positivity rate from January to May 2023 was significantly lower than that during the same period in 2022, which may be related to changes in healthcare-seeking behaviour and testing indications in the early period after NPIs were lifted, rather than representing a true decline in epidemic intensity. Compared with 2022, the epidemic season in 2023 was markedly delayed and prolonged. This phenomenon is consistent with post-pandemic trends observed in southern China [23,30]. Although we could not directly measure climatic factors or population immunity, it is plausible that prolonged suppression of MP transmission during the NPI period led to waning population immunity, creating a larger pool of susceptible individuals, which in turn resulted in a delayed but intense rebound after restrictions were lifted.

This study has several limitations. First, as this was a single-centre retrospective study limited to Chengdu and surrounding areas, the findings may not be generalizable to primary care settings, general hospitals, or other geographic regions. Second, the number of MP tests in 2023 was substantially higher than in 2022, which may reflect changes in healthcare demand, access to testing, or physician testing thresholds. This limits the direct comparability of positivity rates between the two years, the future studies with standardized active surveillance are needed to validate these findings. Third, this study defined MP positivity solely on the basis of a single IgM serological test. IgM antibodies can persist for weeks to months, and the higher positivity rate observed in 2023 may partly reflect accumulated seropositivity from past infections rather than exclusively acute cases. thus, long-term epidemiological surveillance of MP remains necessary in the future. Forth, this study did not capture several variables, including clinical severity, specific respiratory syndrome diagnosis, comorbidities, indication for testing and detailed geographic origin within the catchment area. The absence of these variables limits our ability to perform detailed subgroup analyses and adjust for confounding.

In summary, this single-centre study systematically reviewed the epidemiological changes in MP antibody positivity among children in Chengdu from 2022 to 2023, focusing on comparisons between the pandemic and post pandemic periods. The results demonstrated that use of NPIs influenced both the epidemic intensity and seasonal pattern of MP; following the complete lifting of control measures, the accumulation of susceptible children increased infection risk, with the epidemic peak shifting to autumn and winter. This study provides a baseline reference for local healthcare institutions to improve the early identification and prevention of MP infections. Future studies should be designed as prospective cohorts that systematically collect clinical severity, comorbidities, testing indications, and geographic information to more reliably assess true epidemiological changes in mycoplasma pneumoniae.

## Figures and Tables

**Table 1:** Patient characteristics and detection of *Mycoplasma pneumoniae* between 2022 and 2023.

	2022	2023	χ^2^ Value	*p* Value
Characteristics, (% of total samples received)				
Age				
0–28 days	8 (0.06%)	11 (0.04%)	0.35	0.55
28 days–1 years	1005 (7.14%)	1156 (4.54%)	119	<0.001
1–3 years	2561 (18.2%)	5054 (19.84%)	15.69	<0.001
3–6 years	5926 (42.11%)	10,380 (40.74%)	6.97	<0.01
6–14 years	4326 (30.74%)	7517 (29.50%)	6.58	<0.01
>14 years	248 (1.76%)	1360 (5.34%)	297.2	<0.001
Category				
Outpatient	12,054 (85.65%)	23,302 (91.46%)	322.9	<0.001
Inpatient	2020 (14.35%)	2176 (8.54%)		
Gender				
Boy	7471 (53.08%)	13,059 (51.26%)	12.13	<0.001
Girl	6603 (46.92%)	12,419 (48.74%)		
Total samples	14,074	25,478		

**Table 2:** The overall positive rate of *Mycoplasma pneumoniae* based on age between 2022 and 2023.

Age	2022	2023	χ^2^ Value	*p* Value
0–28 days	2 (25%)	2 (18.18%)	0.13	0.72
28 days–1 years	160 (15.92%)	104 (8.90%)	24.03	<0.001
1–3 years	603 (23.55%)	1261 (24.95%)	1.82	0.18
3–6 years	1436 (24.23%)	2813 (27.10%)	16.1	<0.001
6–14 years	1250 (28.90%)	3176 (42.25%)	209.3	<0.001
>14 years	65 (26.21%)	169 (12.43%)	32.05	<0.001

Data were expressed as the positive number/total tests in that age (%).

**Table 3:** Monthly MP positivity rates in 2022 and 2023.

Month	2022 Positive Tests (%)	2023 Positive Tests (%)	Rate Ratio (2023/2022)	95% CI	*p* Value
January	517 (32.97)	108 (19.05)	0.578	(0.479–0.697)	<0.001
February	325 (24.40)	106 (18.00)	0.738	(0.605–0.900)	0.002
March	323 (22.99)	195 (16.33)	0.71	(0.602–0.838)	<0.001
April	372 (21.99)	292 (17.39)	0.791	(0.687–0.912)	0.001
May	334 (22.91)	222 (16.72)	0.73	(0.622–0.857)	<0.001
June	440 (26.54)	533 (27.49)	1.036	(0.926–1.159)	0.527
July	274 (19.30)	605 (32.35)	1.677	(1.475–1.906)	<0.001
August	236 (27.54)	635 (31.13)	1.131	(0.997–1.283)	0.055
September	87 (24.58)	866 (31.68)	1.289	(1.060–1.566)	0.01
October	191 (28.98)	1407 (34.38)	1.186	(1.040–1.353)	0.01
November	263 (25.99)	1440 (32.45)	1.249	(1.113–1.401)	<0.001
December	153 (23.18)	1111 (36.94)	1.594	(1.363–1.863)	<0.001

(%) = (positive number/total tests in that month) × 100. Rate ratio = 2023 positivity rate/2022 positivity rate. 95% CI = confidence interval for the rate ratio. *p* value from two-proportion z-test (Fisher’s exact test for low counts).

**Table 4:** Multivariable logistic regression analysis of factors associated with *Mycoplasma pneumoniae* seropositivity.

Variable	Reference	Adjusted OR	95% CI	*p* Value
Year				
2023	2022	1.12	(1.063–1.180)	<0.001
Sex				
Male	Female	0.714	(0.682–0.747)	<0.001
Patient type				
Inpatient	Outpatient	1.331	(1.236–1.434)	<0.001
Age group	3–6 years			
29 days–1 year	3–6 years	0.379	(0.331–0.434)	<0.001
1–3 years	3–6 years	0.915	(0.859–0.976)	0.007
0–28 days	3–6 years	0.344	(0.078–1.523)	0.16
6–14 years	3–6 years	1.635	(1.552–1.723)	<0.001
≥14 years	3–6 years	0.356	(0.307–0.413)	<0.001
Month	January			
February	January	0.72	(0.623–0.832)	<0.001
March	January	0.578	(0.505–0.663)	<0.001
April	January	0.575	(0.506–0.654)	<0.001
May	January	0.555	(0.485–0.635)	<0.001
June	January	0.816	(0.722–0.923)	0.001
July	January	0.831	(0.733–0.940)	0.003
August	January	0.972	(0.856–1.104)	0.663
September	January	1.051	(0.925–1.193)	0.446
October	January	1.157	(1.029–1.301)	0.015
November	January	1.009	(0.899–1.133)	0.877
December	January	1.204	(1.065–1.361)	0.003

Note: OR, odds ratio; CI, confidence interval. Age groups: reference = 3–6 years. Sex OR represents male vs. female (OR < 1 indicates lower risk in males, i.e., higher risk in females). Month reference = January.

## Data Availability

The original contributions presented in this study are included in the article. Further inquiries can be directed to the corresponding author.
